# A randomized trial to evaluate the impact of Singapore’s forthcoming Nutri-grade front-of-pack beverage label on food and beverage purchases

**DOI:** 10.1186/s12966-023-01422-4

**Published:** 2023-02-15

**Authors:** Soye Shin, Jyotika Puri, Eric Finkelstein

**Affiliations:** Program in Health Services and Systems Research, Duke-NUS Medical School, Level 4, Singapore, 169857 Singapore

**Keywords:** Nutri-grade labels, Front-of-pack beverage labels, Sugar-sweetened beverages, Diet quality, Online grocery store, Singapore

## Abstract

**Background:**

The epidemic of non-communicable diseases (NCDs) is a growing concern worldwide and Singapore is no exception to this global trend. As part of measures to address this concern, the Singapore government will implement a mandatory color-coded front-of-package (FOP) nutrition label for beverages, called Nutri-Grade (NG), which will complement the existing FOP label, Healthier Choice Symbol (HCS) logos, currently displayed on select food and beverage items. NG grades beverages on a four-point scale, A (healthiest) to D (least healthy), in terms of sugar and saturated fat levels. This study aimed to evaluate the effectiveness of the NG label on nutritional quality of pre-packaged beverages using a fully functional online grocery store.

**Methods:**

We conducted a 2-arm crossover trial involving actual purchases with 138 participants randomly exposed to: 1) Control with HCS logos displayed on qualifying items; 2) Similar to Control except that all beverages displayed the NG label. The effects of the NG label were estimated using a linear mixed-effects model that addresses correlations between repeated measures and accommodates missing data.

**Results:**

We found that the NG label encouraged consumers to choose beverages with higher ratings. This led to a reduction in sugar from beverages purchased by 1.51 g [95% CI: − 2.68, − 0.34] per serving but was not effective at reducing saturated fat purchased (− 0.009 g [95% CI: − 0.22, 0.20]) per serving or improvements in overall diet quality, measured by the weighted (by the number of servings) average Nutri-Score value ranging from 1 to 5 (− 0.024 [95% CI: − 0.13, 0.08]).

**Conclusions:**

Results suggest that the Nutri-Grade label is likely to reduce sugar purchased from beverages. However, to improve overall diet quality in Singapore, additional measures will be needed.

**Trial registration:**

This trial was registered on ClinicalTrials.gov under the identifier: NCT05018026 on 24th August 2021.

**Supplementary Information:**

The online version contains supplementary material available at 10.1186/s12966-023-01422-4.

## Introduction

The epidemic of non-communicable diseases (NCDs) is a growing concern worldwide. NCDs, including but not limited to diabetes, cardiovascular diseases, and cancer, account for 41 million deaths annually, which is equivalent to 71% of global deaths [1]. A large body of literature shows that poor diet quality is a key driver of this global health challenge [2–6]. Of particular concern is excess consumption of sugar, particularly from sugar-sweetened beverages (SSBs) [7, 8]. Their liquid form and rapid digestion make sugar consumption easier and faster compared with solid foods [9–11]. As a result, SSBs are a primary contributor to obesity and NCDs [12].

Singapore is no exception to the global trend of poor diet quality and rising NCDs. The 2018 National Nutrition Survey showed that Singaporean adults consumed an average of 60 g of sugar daily (10 grams above the daily recommended intake), more than half of which came from SSBs [13]. The city-state now has the highest prevalence of diabetes among developed countries [14]. In efforts to address this concern, the Singapore Health Promotion Board (HPB) has taken a series of measures. One is to implement a mandatory front-of-pack (FOP) nutrition label, called Nutri-Grade (NG), that will be implemented in December 2022 [15].

**Fig. 1:**
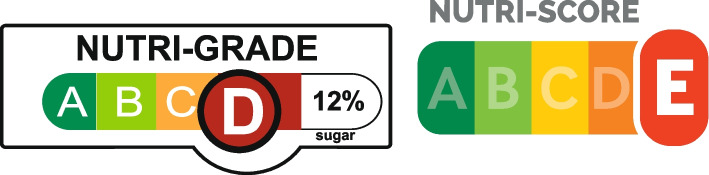
Nutri-Grade label D for a beverage (Left); Nutri-Score label E for a food or beverage (Right)

NG grades freshly-prepared and pre-packaged beverages on a four-point scale, in terms of *sugar and saturated fat levels*, the two primary nutrients of concern for beverages. The grading system works by first assigning each beverage a grade, A (healthiest) to D (least healthy), according to sugar thresholds per 100 ml. Beverages may also be downgraded (but never upgraded) based on saturated fat thresholds per 100 ml. The beverages most likely to be downgraded are dairy beverages, including full cream and flavoured milks. The NG labels will be mandatory only for Grades C and D beverages [16]. In terms of display, the NG label appears similar to France’s Nutri-Score (NS), which has been shown to positively influence diet quality [17–19], although the NG also displays the percentage of sugar in the beverage (see Fig. 1). However, whereas NS applies to both foods and beverages, NG is limited to beverages. The NG label will complement the existing positive Healthier Choice Symbol (HCS) logos currently displayed on select food and beverage items. These labels are voluntary, but most manufacturers include the label on qualifying products. There are currently over 4000 products showing one of 30 HCS logos, spanning across over 100 food categories.

This study aims to evaluate the impact of the NG label on 1) beverage product selection (measured by weighted (by the number of servings) average NG score of beverages purchased, where A = 4, ..., D = 1) and 2) nutritional quality (average sugar, saturated fat, and calories per serving) of beverages purchased using an online grocery platform and shoppers in Singapore. Although NG will be mandatory only for Grade C or D beverages, in this study the full range of grades was displayed as the HCS experience suggests that manufacturers are likely to display the labels on their healthier products.

Although the label is soon to be implemented, the use of the experimental online grocery store allows us to quantify the effects of the label independent of real-world confounders. This includes not only other food policies, including an SSB advertisement prohibition that will be implemented simultaneously with Nutri-Grade, but also other confounders, such as changes in retail prices and employment rates. By using randomization and a control condition, our estimates of the causal effects of the label will inform policymakers of any potential intended and unintended consequences of the labels on food purchases.

Beyond the local relevance, this study contributes to the growing literature on FOP nutrition labelling in general and specifically with regards to color-coded graded labels. The Singapore HPB chose to focus on these labels because they are generally well understood by consumers, effective at promoting healthier food choices [17, 20, 21], and relatively free from unintended consequences such as ‘health halo’ effects [22–24]. However, how this specific approach to color-coded labels performs is ultimately an empirical question that this study will address.

As specified in the Trial Registry (ClinicalTrials.gov), we expect that the labels are likely to shift beverage demand towards healthier beverages, *defined as those with the NG A & B grades*, and improve nutritional quality of beverages purchased. We also tested if the NG labels influence nutritional quality of all foods and beverages (F&B) purchased. This depends on the extent to which diet quality (in terms of sugar and saturated fat) of beverages purchased increases and whether consumers compensate for any healthier beverages purchased by purchasing less healthy foods [25, 26]. Focusing on beverages only also allows for determining whether there may be unintended consequences in terms of additional beverage purchases of nutrients of concern (e.g., sodium) that are not targeted in the NG label. This effort will provide evidence of the potential effectiveness of the NG label in Singapore.

## Methods

### Online grocery store platform: NUSMart

This study takes advantage of the NUSMart, online experimental grocery store, developed by the research team. NUSMart was designed for research purposes to mimic actual web-based grocery stores in Singapore in both look and feel. To populate the store with local products and prices, we collaborated with one of the largest grocery retailers in Singapore, called FairPrice. FairPrice provided access to their Application Programming Interfaces (APIs) for real time stock availability, pricing, and food delivery by their drivers for confirmed orders. Our store contained 2500 of their most popular food and beverage products, classified into 23 categories. All product listings included pictures of the items, product brand, description, prices, and Nutritional Facts Panel (NFP) information available via click-through. We have successfully used this store in seven publications (see https://nusmartbulletin.wordpress.com/) with over 2600 participants. In each study, participants provided feedback on the shopping experience, which we then reviewed and incorporated into future iterations of the store when feasible.

### Experimental design and procedure

**Fig. 2:**
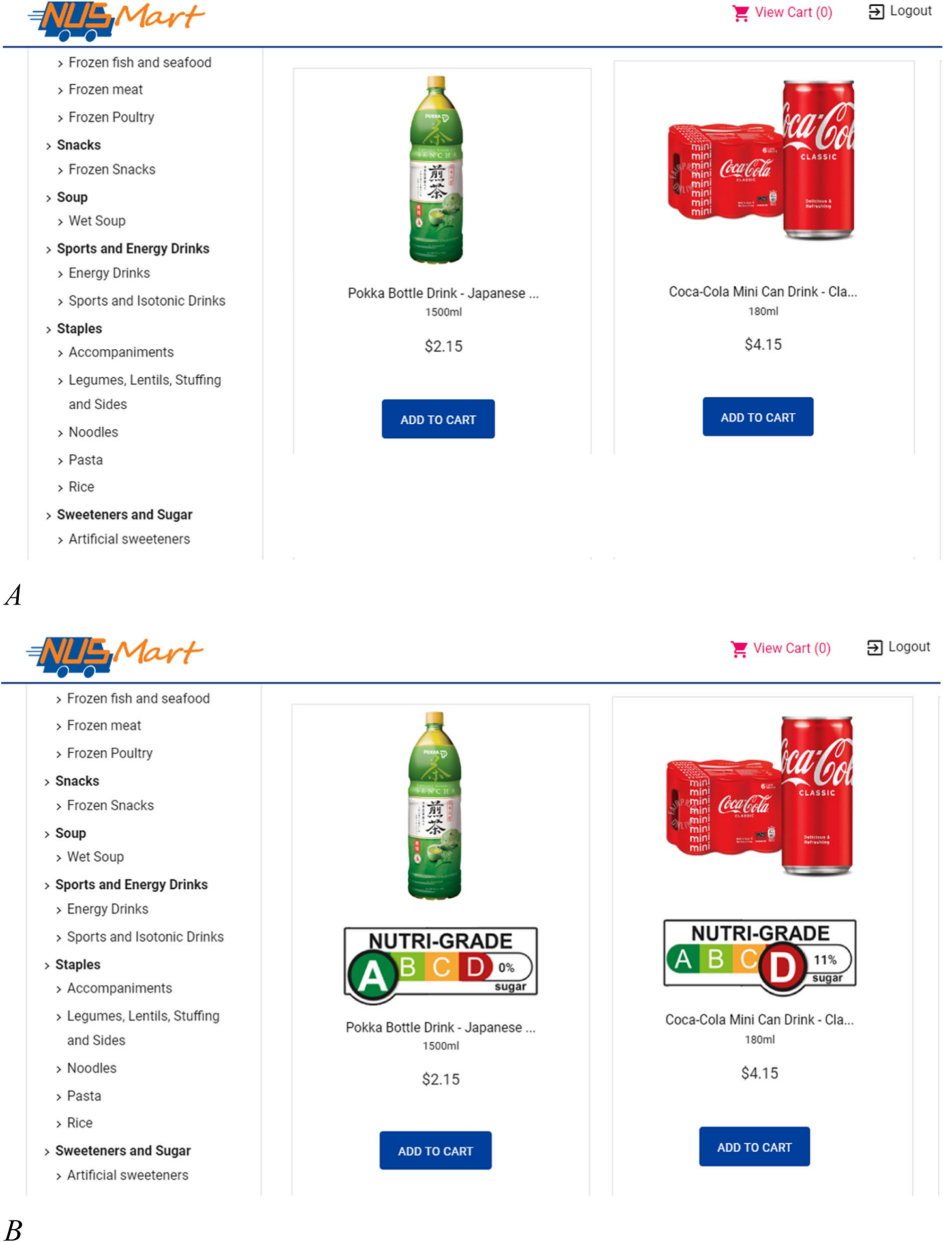
Two versions of NUSMart with example products: (**A**) control condition and (**B**) NG condition

To evaluate the NG labels, we employed a two-arm crossover design where participants shopped once on the following two NUSMart versions in random order (see Fig. 2). The crossover design allows for a smaller sample size than a between-subjects design although it may suffer from contamination if learning takes place that influences subsequent shops. We chose the crossover design given our limited budget for this effort. Each participant consented to make two shops 1 week apart. The first shop for each participant was randomly allocated to either Control or NG, with the second shop being the other arm. We imposed this one-week gap and randomization to minimize carryover effects.

- Control condition: A default NUSMart that mirrored a conventional web-grocery store in Singapore where qualifying healthier products displayed the HCS logos.
- NG condition: Similar to Control condition except that all beverages displayed the appropriate NG label. Because this label is new to Singaporean shoppers, prior to this shop participants were required to watch a video explaining the new label and how it can be used to improve diet quality.

Participants were asked to purchase weekly groceries similar to their typical grocery shopping experience, with a minimum spend of Singapore Dollar (SGD) 50 for each shop. The minimum spend was determined based on the average weekly household expenditure on food & non-alcoholic beverages reported in the 2017/18 Singapore Household Expenditure Survey and by accounting for households with only 1 or 2 members and smaller varieties on our store than those available in FairPrice, our food delivery partner. Given that the NG labels are displayed on beverages only, we also required participants to buy at least one beverage product at each shop. This ensured they were exposed to the new labels.

Participants were informed that in each of the shops there was a 50% chance that they would have to purchase their order, which would be determined by spinning a “Wheel of Purchase” immediately after checkout. When the “Wheel of Purchase” indicated that the shop was selected for purchase and delivery, the corresponding grocery cart was transferred to the FairPrice webstore where the participant managed payment and delivery via his/her FairPrice account. The products were delivered to the participant’s homes by FairPrice. This approach reduced logistical challenges while also minimizing biases that come with hypothetical shopping.

A power calculation revealed that 108 participants were required to detect a 0.3 standardized effect size difference in our primary outcome of weighted (by the number of servings) average grams of sugar per serving, the primary target of the NG label, assuming a power of 0.8, a significance level of 0.05, a correlation of 0.5 in purchases across two shops, and a 20% attrition rate. The standardized effect size of 0.3 was applied based on results of prior labelling studies [23, 27–29] and Cohen’s D Rule of Thumb that 0.3 is between a small (0.2) and medium effect size (0.5). Between September and December 2021, participants were recruited through Facebook and Instagram advertisements as well as email blasts disseminated by FairPrice’s marketing team. Eligibility criteria were Singapore residents aged 21 years or older who were the primary grocery shoppers in their household and shopped at least once a week. Additionally, they should either have an existing FairPrice online account or be willing to create one for the study.

Interested participants were directed to a Participant Information Sheet (PIS) and consent document. The PIS form contained the information about the study procedure and requirements but did not disclose the intervention. Once participants consented, they were directed to a baseline survey collecting demographic and health information. For the latter, we asked if any of their household members had been diagnosed with diet-related health conditions.

Those who completed the baseline survey were randomized by a computer program and officially enrolled in the study. Participants were then informed by email and asked to complete two grocery shops 1 week apart. Weekly reminders were sent for those who did not complete the required shops. Upon completion of their second shop, participants were asked to fill in a short post-study survey asking if they understood/used the NG label for beverage purchases and were willing to use the label if it were displayed on food products. Participants who completed both shops and the surveys received SGD 50 via digital wallet applications for their participation. All procedures were approved by the National University of Singapore Institutional Review Board (Reference Code: NUS-IRB-2021-128). The trial was registered on ClinTrials.gov (Identifier: NCT05018026, Registered on: 24th August 2021) and can be accessed online.

### Statistical analysis

#### Outcome variables

To test the effect of the NG label on beverages purchased we quantified the weighted (by the number of servings) average NG score of beverages purchased, after recoding each letter to a number value: A = 4, B = 3, C = 2, and to D = 1. For all foods and beverages, we relied on the weighted (by the number of servings) average Nutri-Score (NS) value given that it is a valid measure of overall diet quality [30–34] and can capture changes in nutritional quality of grocery baskets when shoppers compensate healthier beverages purchased for less healthy foods. Relying on the British Food Standard Agency Nutrient Profiling systems [35–37], NS assesses overall diet quality of each product with a grade between A (healthiest) and E (least healthy) based on the levels of seven nutritional components (four negative components: energy, sugar, sodium, and saturated fat; and three positive components: a percentage of fruits and vegetables, protein, and dietary fiber) per 100 g or 100 ml. Similar to the weighted average NG score, we obtained the weighted average NS of the grocery baskets after recoding each NS grade to a numeric value, with NS A = 5 to E = 1.

We also assessed nutritional quality of beverages and F&B purchased, in terms of each nutritional aspect of interest. The nutrient-specific outcomes include the weighted (by the number of servings) average grams of sugar per serving (primary), average saturated fat per serving, calories per serving, and total sugar, saturated fat, and calories of the grocery baskets. Measured per-serving and total changes allows us to test whether the label may improve diet quality per serving but increase total number of servings, nutrients, and calories purchased [22, 38].

#### Analysis

We employed a linear mixed-effects model that not only addresses correlations between repeated measures but accommodates missing data that can result when participants drop out before study completion [39–41].

Accordingly, our base model was:

$${Y}_{ijk}={\beta}_0+{\beta}_1{NG}_{ijk}+{X}^{\prime}\gamma +{\theta}_j+{\mu}_i+{\varepsilon}_{ijk}$$

where *Y*_*ijk*_ is an outcome variable of interest observed for participant *i* in sequence *k* (*k* =1 (Control-NG), 2 (NG-Control)) at period *j* (*j* =1, 2) and *NG*_*ijk*_ is an indicator that is equal to 1 when participant *i* in sequence *k* shops on the NG store at period *j*. Accordingly, the constant term *β*_0_ represents the mean outcome value in the control condition while the coefficient *β*_1_ represents the incremental effect on the outcome due to the NG label. *X* is a vector of covariates that include age, and dummies for female, high education level (university degree and above), high income (monthly household income of SGD 10,000 and above), along with two indicators for health status; whether the respondent’s BMI is high (i.e., BMI ≥ 25 for overweight or obese) and whether all household members have no diet-related health conditions, including obesity, diabetes, high blood cholesterol, high blood pressure, heart disease/stroke, gastrointestinal disorders, and kidney ailments. The covariates were chosen based on the previous evidence of their association with food choices [42–45]. The period fixed effect (*θ*_*j*_), a subject-specific random effect (*μ*_*i*_) capturing the variance in outcomes within individuals and the error term (*ε*_*ijk*_) were included.

We first limited the data to beverages and estimated this model for the weighted average NG score as well as the nutrient-specific outcomes. Then, using the entire grocery basket, we ran the identical models but replacing the weighted average NG with the weighted average NS. For all analyses, we used a *p*-value < 0.05 to determine the statistical significance of the coefficients.

## Results

### Sample

**Fig. 3:**
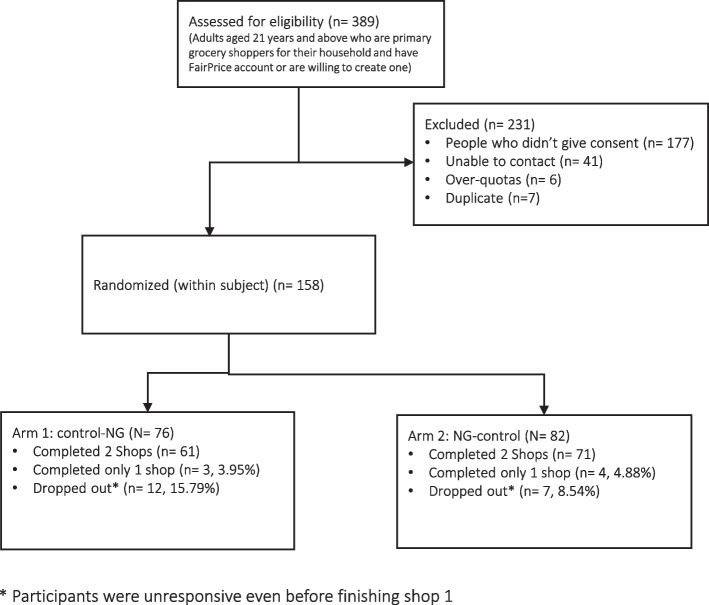
Participant Flow Chart

Figure 3 presents an overview of the participant flow and randomization. Among 389 individuals who filled in the screener, 158 were eligible, consented to participate and were randomized into one of the two sequences (Control-NG or NG-Control). Of the 158 participants enrolled, 19 (12%) dropped out before completing the first shop and 7 (4%) completed only one shop (non-completers). One participant was excluded for not purchasing a single beverage product in one of the shops. This left 138 participants with 269 unique shops for analysis, which were obtained from 131 completers and 7 non-completers. Our estimating model handled the issue of missing data for those 7 participants with the assumption that the data is missing at random. Including or excluding the non-completers did not change our results.

Table 1 describes the demographics of the sample. The mean age was 43 years (SD = 10) with 69% being female. The sample was generally highly educated with 78% achieving “University degree or above”. The portion of the participants whose household monthly income was “Singapore dollar (SGD) 10,000 and over” was 36%. The majority of the participants (67%) lived in government housing with three to five rooms. Average BMI was 24 *kg*/*m*^2^ (SD = 5) with 38% having a BMI of 25 *kg*/*m*^2^ and over (i.e., overweight/obese), and 34% reported to have no household member with a diagnosis of the included diet-related health conditions.

**Table 1:** Summary statistics of demographics for full sample

		N (%)/ Mean (SD)
Age, Mean (SD)		43.21 (9.99)
Gender, N (%)		
	Female	94 (68.12%)
	Male	44 (31.88%)
Education Level, N (%)		
	Post-secondary & vocational training	20 (14.49%)
	Other diploma & professional qualification	12 (8.70%)
	University & above	106 (76.81%)
Income Level (monthly in SGD), N (%)		
	Below $2000	5 (3.62%)
	$2000-$5999	25 (18.12%)
	$6000-$9999	41 (29.71%)
	$10,000 and over	50 (36.23%)
	Do not know income	1 (0.73%)
	Prefer not to say income	16 (11.59%)
Housing type, N (%)		
	HDB/JTC Flat (3 room)	16 (11.59%)
	HDB/JTC Flat (4-5 room)	76 (55.07%)
	Condominium/Private Flat	36 (26.09%)
	Bungalow/Semi-attached/terrace house	10 (7.25%)
BMI (w *eight*(*kg*) / *height*^2^(*m*^2^)), Mean (SD)		24.29 (4.87)
Household member(s) having medical history, N (%)		
	Has a health condition	90 (65.22%)
	No health condition	48 (34.78%)
Observations		138

*HDB* (Housing and Development Board): government housing, *JTC* (Jurong Town Corporation): government housing, *SGD* Singapore Dollars

As a whole, the mean grocery spending was 71 SGD (SD = 22). The proportion of beverages selected for purchase was 29% in terms of quantities and 27% in terms of total spending. Responses to the post-survey questions revealed that over 95% of the participants understood the label by correctly matching the NG D label to a soft drink product, as opposed to a bottled water product, and 89% responded ‘Yes’ when asked ‘If these labels were displayed on *food products*, would you use them to make your food purchases?’

### The effects of the Nutri-grade label on diet quality of beverages purchased

Table 2 presents the results of estimating the base model for 1) beverage purchasing patterns (weighted average NG) and 2) nutritional quality of the beverages purchased.

**Table 2:** Regression results showing the effects of Nutri-Grade and other covariates on diet quality: beverages (*n* = 269)

	Average NG	Sugar (g) per serving	Saturated fat (g) per serving	Calories (kcal) per serving	Total sugar (g)	Total saturated fat (g)	Total calories
NG	0.19^***^	−1.51^**^	−0.0092	−3.42	−21.8	1.11	−32.3
	[0.068, 0.31]	[−2.68, − 0.34]	[− 0.22, 0.20]	[− 11.9, 5.09]	[−59.4, 15.8]	[− 5.19, 7.41]	[− 320.3, 255.6]
Age	0.0023	− 0.069	0.0025	− 0.28	−4.41^**^	− 0.30	− 29.6^**^
	[− 0.0092, 0.014]	[− 0.17, 0.035]	[− 0.016, 0.021]	[− 0.97, 0.41]	[−8.67, − 0.14]	[− 0.76, 0.17]	[−55.7, − 3.43]
Female	− 0.20^*^	1.26	0.031	5.07	− 65.2	− 2.93	− 427.4
	[− 0.43, 0.032]	[− 0.86, 3.37]	[− 0.34, 0.40]	[− 8.93, 19.1]	[− 151.6, 21.1]	[−12.4, 6.55]	[− 956.9, 102.1]
High edu	0.14	−1.40	0.39^*^	0.68	− 111.9^**^	−1.86	− 676.0^**^
	[− 0.13, 0.41]	[−3.86, 1.06]	[− 0.039, 0.82]	[− 15.6, 17.0]	[− 212.3, − 11.5]	[− 12.9, 9.20]	[− 1292.8, − 59.3]
High income	0.15	− 0.59	0.05	−2.51	−13.8	− 0.84	− 19.3
	[−0.078, 0.38]	[− 2.65, 1.46]	[− 0.31, 0.41]	[−16.2, 11.1]	[− 98.0, 70.3]	[− 10.1, 8.41]	[− 535.5, 497.0]
High BMI	−0.24^**^	2.63^**^	0.10	9.62	86.5^**^	0.67	466.2^*^
	[−0.46, − 0.0093]	[0.58, 4.69]	[− 0.26, 0.46]	[− 3.99, 23.2]	[2.66, 170.3]	[− 8.54, 9.89]	[−48.1, 980.6]
No health condition	− 0.37^***^	3.25^***^	0.27	11.2	90.0^**^	5.12	299.3
	[−0.61, − 0.14]	[1.11, 5.39]	[− 0.10, 0.65]	[−2.96, 25.5]	[2.50, 177.4]	[−4.51, 14.7]	[− 237.7, 836.3]
Constant	2.76^***^	10.9^***^	0.19	66.6^***^	516.5^***^	34.2^**^	3542.2^***^
	[2.09, 3.43]	[4.78, 16.9]	[−0.87, 1.26]	[26.2, 107.0]	[268.1, 764.9]	[6.77, 61.5]	[2016.5, 5067.8]
Period fixed effect (*θ*_*j*_)	−0.093	0.33	0.14	3.64	5.47	0.69	17.3
	[−0.22, 0.030]	[−0.84, 1.51]	[− 0.078, 0.35]	[−4.88, 12.2]	[−32.1, 43.1]	[−5.61, 7.00]	[− 270.7, 305.4]
Ln (*σ*_*μ*_)	− 0.67^***^	1.51^***^	− 0.26^**^	3.34^***^	5.32^***^	2.86^***^	7.04^***^
	[−0.86, − 0.48]	[1.31, 1.71]	[− 0.48, − 0.036]	[3.10, 3.57]	[5.17, 5.48]	[2.56, 3.15]	[6.85, 7.24]

Ln (*σ*_*μ*_) represents the logarithm of the standard deviation of constant (subject-specific random effect; *μ*_*i*_);* *p* < 0.1, ** *p* < 0.05, *** *p* < 0.01

*NG* Nutri-Grade

Consistent with our primary hypothesis, the NG label was effective at increasing purchases of NG A and B beverages, relative to the control condition. The weighted average NG score was higher by 0.19 [95% CI: 0.068,0.31, *p*-value: 0.002] in the NG condition, which was equivalent to a 7% difference compared with the control (2.76).

Although there was a demand shift toward healthier beverages as defined by the average NG score, the effects were limited to sugar. Compared with the control condition, sugar purchased from beverages was lower by 1.5 g per serving [95% CI: − 2.68, − 0.34, *p*-value: 0.012] in the NG condition. However, no statistical difference in saturated fat [− 0.009 g; 95% CI: − 0.22,0.20] and calories per serving [− 3.4 kcal; 95% CI: − 11.9, 5.1] was observed between conditions. The negative (but not statistically significant) coefficients on total energy and most nutrients (except for total saturated fat having a positive coefficient) suggest that the NG label did not induce shoppers to purchase more healthier beverages relative to the control condition.

Among the covariates, shoppers who reported to have no diet-related health conditions among their household members purchased more NG A and B beverages. By contrast, high BMI shoppers tended to buy more high sugar beverages. Being older and more educated were negatively associated with the total calories of the grocery baskets. As an exploratory analysis, we report the heterogeneous effects by education, income level, and the two health-status indicators for beverages in Supplementary Table A1 in Additional file 1. We found that shoppers with high income (i.e., SGD 10,000 and above) had a greater mean reduction in sugar per serving in response to the NG label (*p*-value: 0.029).

Given no effect on saturated fat, we conducted an exploratory analysis to obtain further insight into this outcome. We classified beverage subcategories into dairy (e.g., fresh milk, yoghurt drinks) and non-dairy (e.g., fruit juices, carbonated soft drinks) groups given that saturated fat content is relatively more important among the former group while sugar is for the latter group. We then tested whether the average sugar and saturated fat per serving, and proportion of *less healthy (*i.e.*, NG C & D)* beverages (in terms of servings) significantly differed by condition for each subgroup.

Among dairy products, we found no difference in sugar per serving between the conditions but slightly lower saturated fat per serving in the control condition (*p* = 0.080). Among non-dairy products, both per serving sugar and saturated fat were lower in the NG condition although *p*-values were above the conventional cutoff of 0.05 (*p* = 0.07 for sugar and p = 0.08 for saturated fat). We further compared the proportions of NG C & D beverages between the conditions. While for non-dairy, the NG condition had a lower proportion of less healthy beverages than the control (*p* = 0.007), there was no statistical difference among dairy beverages between the conditions.

### The effects of the Nutri-grade label on diet quality of foods and beverages purchased

Results from estimating the main model for all F&B are shown in Table 3. Note that we used the weighted average NS value to assess the effect of the label on overall diet quality of the food and beverage products in the grocery baskets. The results for nutrients that are not the target of the NG label but accounted for in the NS algorithm are reported in Supplementary Table A2 in Additional file 1. Contrast to the results for beverages only, none of the outcomes statistically changed in response to the NG label. These results suggest that the labels did not encourage increased purchases of other nutrients of concern either due to compensatory behavior or because the label focused on only sugar and saturated fat. As for the covariates, we found shoppers with high BMI and the indicator of no diet-related health condition among household were negatively associated with overall diet quality, measured by the weighted average NS (*p*-value: 0.013 for High BMI; *p*-value: 0.035 for No health condition), which echoes the results for the weighted average NG and sugar per serving among beverages. Individuals with high education attainment purchased less total sugar (*p*-value: 0.044).

**Table 3:** Regression results showing the effects of Nutri-Grade and other covariates on diet quality: foods and beverages (*n* = 269)

	Average NS	Sugar (g) per serving	Saturated fat (g) per serving	Calories (kcal) per serving	Total sugar (g)	Total saturated fat (g)	Total calories
NG	− 0.024	0.33	− 0.043	2.39	−60.8	−34.6	− 419.6
	[−0.13, 0.080]	[− 0.73, 1.40]	[− 0.42, 0.33]	[−17.9, 22.6]	[− 441.0, 319.4]	[− 126.5, 57.2]	[− 4091.6, 3252.5]
Age	0.0022	− 0.0027	0.0087	1.33^*^	5.03	5.43	311.8^*^
	[−0.0092, 0.013]	[−0.078, 0.073]	[− 0.016, 0.033]	[− 0.042, 2.71]	[−29.5, 39.6]	[−2.84, 13.7]	[−18.8, 642.4]
Female	− 0.15	0.46	0.14	5.26	185.4	85.0	2084.2
	[−0.38, 0.082]	[−1.07, 2.00]	[−0.35, 0.64]	[−22.6, 33.1]	[−514.8, 885.6]	[−82.6, 252.5]	[− 4612.9, 8781.3]
High edu	0.21	−0.56	0.27	31.6^*^	− 838.6^**^	−44.5	− 1846.1
	[−0.057, 0.48]	[−2.35, 1.23]	[−0.31, 0.85]	[− 0.97, 64.1]	[− 1654.2, − 23.1]	[− 239.7, 150.7]	[− 9647.0, 5954.8]
High income	0.019	−0.39	− 0.15	−31.7^**^	479.4	61.3	− 372.6
	[−0.20, 0.24]	[−1.88, 1.11]	[−0.63, 0.33]	[−58.8, − 4.47]	[− 203.3, 1162.1]	[− 102.0, 224.7]	[− 6901.8, 6156.7]
High BMI	− 0.28^**^	0.25	0.31	−8.35	221.5	43.8	3475.8
	[−0.50, − 0.058]	[−1.24, 1.74]	[− 0.17, 0.80]	[−35.5, 18.8]	[− 458.7, 901.7]	[− 119.0, 206.6]	[− 3030.3, 9981.9]
No health condition	− 0.25^**^	0.88	0.14	−8.03	162.0	8.54	1169.0
	[−0.48, − 0.018]	[− 0.68, 2.44]	[−0.37, 0.64]	[−36.4, 20.3]	[− 548.1, 872.2]	[−161.4, 178.5]	[− 5623.5, 7961.6]
Constant	2.75^***^	9.14^***^	1.30^*^	66.2	1948.1^*^	122.0	9532.0
	[2.09, 3.41]	[4.70, 13.6]	[−0.14, 2.74]	[−14.4, 146.9]	[−69.4, 3965.6]	[− 360.8, 604.8]	[− 9765.6, 28,829.5]
Period fixed effect (*θ*_*j*_)	0.16^***^	−0.87	−0.048	6.72	− 191.4	−43.0	− 621.7
	[0.051, 0.26]	[−1.94, 0.20]	[−0.42, 0.33]	[−13.6, 27.0]	[− 571.8, 189.1]	[− 134.9, 48.9]	[− 4296.0, 3052.5]
Ln (*σ*_*μ*_)	− 0.62^***^	0.98^***^	− 0.31	3.81^***^	7.33^***^	5.89^***^	9.58^***^
	[−0.78, − 0.46]	[0.65, 1.31]	[− 0.79, 0.17]	[3.42, 4.20]	[7.13, 7.52]	[5.69, 6.09]	[9.38, 9.78]

Ln (*σ*_*μ*_) represents the logarithm of the standard deviation of constant (subject-specific random effect; *μ*_*i*_);* *p* < 0.1, ** *p* < 0.05, *** *p* < 0.01

*NG* Nutri-Grade, *NS* Nutri-Score

## Discussion

This study assessed the effectiveness of Singapore’s upcoming FOP beverage label, Nutri-Grade (NG), on nutritional quality of products purchased using an experimental online grocery store. For beverages, we found that the weighted (by the number of servings purchased) average NG score (ranging from 1 (D) to 4 (A)) was 7% higher in the NG label condition compared to control, suggesting that the label positively influenced shoppers’ beverage purchasing patterns. This increase appears to be driven entirely due to sugar reduction. The NG label reduced per serving sugar purchased by 1.5 g (equivalent to about 2.9 g of sugar/330 ml can). The sugar reduction is small but could have a meaningful effect over time given that Singaporeans consume an average of 33 g of sugar from beverages daily [14, 46]. Our findings about the NG label’s effectiveness are consistent with the evidence on the impact of color-coded graded labels [20, 21, 47]. Although in the FOP labelling literature nutrient-specific outcomes were less commonly measured than purchasing intentions and behavior, the NG’s effect on sugar reduction from beverages purchased is slightly larger than the average effect size of other FOP labels on sugar reduction (0.33 g per 100 g for beverages) reported by a review article [47]. Given the heterogeneity in sample and study designs, however, the direct comparisons should be made with caution.

The NG label was not effective at reducing saturated fat (g) purchased from beverages, despite it being a target nutrient of the NG algorithm. There are several possible explanations for this result. First, the underlying NG algorithm largely focuses on sugar. Beverages with high sugar content receive NG C or D grades even if they are low in saturated fat. This means that consumers switching out of these beverages toward healthier products could actually *increase* saturated fat intake, whereas the converse is not true. Second, most beverages purchased in our study and in reality do not contain saturated fat, or contain only modest quantities, thus the ability to influence this nutrient through labelling policy is limited. Finally, beverages containing saturated fat such as milk could be perceived as less discretionary [48], therefore they may be less willing to give up these purchases due to new information on their health content. Future studies could further explore these hypotheses.

When expanded to the entire shopping basket, we found no difference in overall diet quality. This likely results because beverages only accounted for a small proportion of products purchased and we found no evidence that healthier beverage purchases led consumers to substitute toward less healthy foods. Regardless, the fact that the label did not influence overall diet quality reveals the limits of a label focusing on beverages only. Given Singapore’s goal to reduce the incidence of diabetes and other NCDs, these results suggest that additional policies will be needed.

Singapore has long applied the Healthier Choice Symbol (HCS) logo on packaged food and beverage products. However, the effectiveness of this and other positive FOP labels have been questioned, largely because they do not help to identify the least healthy products, which are highest in sugar and calories [23]. HCS also includes multiple claims for different nutrients, which could confuse consumers [22, 39]. This confusion may only get worse with the addition of NG. As a result, it may be appropriate to implement a unifying NG or NS-type label for all foods and beverages. This statement is bolstered by the fact that 89% of the participants in this study stated that they would use an NG type label if it were displayed on food products and that prior experimental research has already shown NS to be effective at improving diet quality of the shopping basket among shoppers in Singapore [18].

### Limitations

This study has many strengths, including taking advantage of a fully functional online grocery store integrated into a commercial retailer that enabled seamless payment and delivery of a subset of purchases. However, it is not without limitations. Although we included a one-week washout period between shops and randomized the order of the conditions, we cannot rule out the possibility that these eliminated all possible carryover effects from one shop to another. A supplemental analysis that tested for ordering effect (see Supplementary Table A3 in Additional file 1) cannot reject the null hypothesis of no carryover effects, but we may not have sufficient power for such a test. However, if such effects existed, it most likely would have biased the results in favor of the Control, as shoppers may have remembered the NG scores in subsequent shops. This suggests our results may be conservative. Second, it was limited to an experimental grocery store setting which may not match actual shopping behavior over repeated shops. It was also limited to pre-packed products only, yet 20% of beverage consumption among Singaporeans is from freshly-prepared products [46]. Although online grocery shopping has become increasingly popular as a result of the Covid-19 pandemic [49, 50], we acknowledge that our sample disproportionally consisted of higher income populations and hence, the study results may not generalize to the general population. Finally, this study focused only on consumer responses to the label, however, suppliers too may respond. This could be through reformulation or price changes that could also influence diet quality, albeit in unknown directions. Future studies using real purchase data after label implementation will provide insight into the long-run effects of the label. Despite these shortcomings, our findings provide insights about the likely effectiveness of the upcoming Singapore beverage label.

## Conclusions

In conclusion, results suggest that the Nutri-Grade label is likely to reduce sugar purchased from beverages. However, to improve overall diet quality in Singapore, additional measures will be needed.

## Supplementary Information

**Additional file 1.** Supplementary tables including heterogeneous analysis results (A1) the effect of NG on other nutrients of interest (A2), and carryover effect testing results (A3).

**Additional file 2.** CONSORT checklist. Description of data: Additional file 1 is a checklist of information to include when reporting a randomized trial.

**Additional file 3.** TIDieR checklist. Description of data: Additional file 2 is a checklist for intervention description and replication.

## Data Availability

The datasets used and/or analyzed during the current study are available from the corresponding author on reasonable request.

## Abbreviations

NG: Nutri-Grade
NCD: Non-Communicable Diseases
SSB: Sugar-Sweetened Beverages
HPB: Health Promotion Board
FOP: Front-Of-Pack
HCS: Healthier Choice Symbol
BMI: Body Mass Index
F&B: Food and Beverage
API: Application Programming Interface
NFP: Nutrition Facts Panel
SGD: Singapore Dollar
PIS: Participant Information Sheet
NS: Nutri-Score
