# Supplementary material for: A randomized trial to evaluate the impact of Singapore’s forthcoming Nutri-grade front-of-pack beverage label on food and beverage purchases
Source: Int J Behav Nutr Phys Act. 2023 Feb 15;20:18. doi: 10.1186/s12966-023-01422-4 (PMC9930047; doi:10.1186/s12966-023-01422-4)
Supplement: Supplementary file 1 — Additional file 1. Supplementary tables including heterogeneous analysis results (A1) the effect of NG on other nutrients of interest (A2), and carryover effect testing results (A3). [file 12966_2023_1422_MOESM1_ESM.docx]

**Additional File 1: Supplementary Tables**

**Table A1: Regression Results Showing Heterogeneous Effects of Nutri-Grade by Other Covariates on Diet Quality for Beverages**

|  | Average NG | Sugar (g) per serving | Saturated fat (g) per serving |
| --- | --- | --- | --- |
| NG | 0.25 | -1.53 | 0.26 |
|  | [-0.061, 0.55] | [-4.40, 1.33] | [-0.27, 0.80] |
|  |  |  |  |
| Age | 0.0024 | -0.070 | 0.0025 |
|  | [-0.0091, 0.014] | [-0.17, 0.035] | [-0.016, 0.021] |
|  |  |  |  |
| Female | -0.20^*^ | 1.25 | 0.026 |
|  | [-0.43, 0.031] | [-0.86, 3.37] | [-0.35, 0.40] |
|  |  |  |  |
| High edu | 0.16 | -1.34 | 0.48^*^ |
|  | [-0.15, 0.47] | [-4.19, 1.50] | [-0.023, 0.99] |
|  |  |  |  |
| High income | 0.056 | 0.79 | 0.15 |
|  | [-0.21, 0.32] | [-1.62, 3.19] | [-0.28, 0.58] |
|  |  |  |  |
| High BMI | -0.18 | 1.81 | 0.22 |
|  | [-0.44, 0.087] | [-0.60, 4.21] | [-0.21, 0.65] |
|  |  |  |  |
| No health condition | -0.28^**^ | 2.30^*^ | 0.24 |
|  | [-0.55, -0.0074] | [-0.19, 4.79] | [-0.20, 0.69] |
|  |  |  |  |
| High edu $\times$ NG | -0.021 | -0.28 | -0.17 |
|  | [-0.32, 0.28] | [-3.09, 2.52] | [-0.69, 0.35] |
|  |  |  |  |
| High income $\times$ NG | 0.18 | -2.71^**^ | -0.20 |
|  | [-0.080, 0.44] | [-5.14, -0.28] | [-0.65, 0.25] |
|  |  |  |  |
| High BMI $\times$ NG | -0.12 | 1.66 | -0.23 |
|  | [-0.38, 0.15] | [-0.81, 4.13] | [-0.69, 0.22] |
|  |  |  |  |
| No health condition $\times$ NG | -0.18 | 1.80 | 0.064 |
|  | [-0.44, 0.086] | [-0.66, 4.27] | [-0.39, 0.52] |
|  |  |  |  |
| Constant | 2.73^***^ | 10.9^***^ | 0.058 |
|  | [2.05, 3.42] | [4.66, 17.1] | [-1.04, 1.16] |
| Period fixed effect ($\theta_{j}$) | -0.10 | 0.50 | 0.12 |
|  | [-0.23, 0.022] | [-0.66, 1.67] | [-0.092, 0.34] |
| Ln ($\sigma_{\mu})$ | -0.67^***^ | 1.53^***^ | -0.24^**^ |
|  | [-0.86, -0.48] | [1.33, 1.72] | [-0.46, -0.027] |

Note: Observations are 269. Ln ($\sigma_{\mu})$ represents the logarithm of the standard deviation of constant (subject-specific random effect; $\mu_{i}$); * p<0.1, ** p<0.05, *** p<0.01

NG: Nutri-Grade

**Table A2: Regression Results Showing the Effects of Nutri-Grade on Other Nutrients: Foods and Beverages (n=269)**

|  | Sodium (mg) per serving | Fiber (g) per serving | Protein (g) per serving | Total sodium (mg) | Total fiber (g) | Total protein (g) |
| --- | --- | --- | --- | --- | --- | --- |
| NG | -2402.1 | -0.090 | -0.031 | -561768.6 | -7.25 | -34.6 |
|  | [-7706.4, 2902.2] | [-0.22, 0.042] | [-0.62, 0.55] | [-1615281.5, 491744.2] | [-25.8, 11.3] | [-128.9, 59.6] |
|  |  |  |  |  |  |  |
| Age | 281.3^*^ | -0.0020 | 0.017 | 71340.8^**^ | 1.16^*^ | 9.17^**^ |
|  | [-7.79, 570.4] | [-0.012, 0.0077] | [-0.031, 0.065] | [13919.9, 128761.8] | [-0.16, 2.48] | [2.16, 16.2] |
|  |  |  |  |  |  |  |
| Female | -4152.3 | 0.054 | -0.38 | -921608.4 | -0.58 | -35.5 |
|  | [-10010.4, 1705.7] | [-0.14, 0.25] | [-1.35, 0.58] | [-2085100.8, 241884.0] | [-27.3, 26.1] | [-177.7, 106.6] |
|  |  |  |  |  |  |  |
| High edu | 5826.0^*^ | 0.12 | 1.26^**^ | 1158272.2^*^ | 14.2 | 174.0^**^ |
|  | [-1031.8, 12683.8] | [-0.11, 0.35] | [0.14, 2.39] | [-203780.4, 2520324.8] | [-17.0, 45.5] | [8.12, 339.9] |
|  |  |  |  |  |  |  |
| High income | -4188.3 | -0.15 | -0.71 | -855940.3 | -7.99 | -24.5 |
|  | [-9904.3, 1527.8] | [-0.35, 0.039] | [-1.65, 0.23] | [-1991230.4, 279349.7] | [-34.1, 18.1] | [-163.1, 114.2] |
|  |  |  |  |  |  |  |
| High BMI | 3972.1 | -0.096 | -0.38 | 761747.5 | -5.38 | -21.0 |
|  | [-1733.2, 9677.4] | [-0.29, 0.096] | [-1.32, 0.56] | [-371407.3, 1894902.3] | [-31.4, 20.6] | [-159.2, 117.2] |
|  |  |  |  |  |  |  |
| No health condition | -509.6 | -0.19^*^ | 0.072 | 33618.1 | -11.8 | 26.5 |
|  | [-6476.3, 5457.0] | [-0.39, 0.012] | [-0.91, 1.05] | [-1151444.2, 1218680.3] | [-38.9, 15.4] | [-117.9, 170.9] |
|  |  |  |  |  |  |  |
| Constant | -11734.3 | 0.80^***^ | 3.32^**^ | -2976629.1^*^ | 43.2 | 142.2 |
|  | [-28794.4, 5325.8] | [0.23, 1.37] | [0.53, 6.11] | [-6365014.4, 411756.3] | [-34.1, 120.5] | [-268.5, 553.0] |
| Period fixed effect ($\theta_{j}$) | 3329.1 | 0.013 | 0.44 | 584622.1 | -8.54 | 14.4 |
|  | [-1979.5, 8637.7] | [-0.12, 0.15] | [-0.15, 1.02] | [-469739.5, 1638983.7] | [-27.1, 10.0] | [-79.9, 108.8] |
| Ln ($\sigma_{\mu})$ | -5.24 | -1.02^***^ | 0.67^***^ | 1.20 | 3.84^***^ | 5.57^***^ |
|  | [-1081.0, 1070.5] | [-1.31, -0.73] | [0.44, 0.90] | [-2.83, 5.22] | [3.50, 4.17] | [5.28, 5.86] |

Note: Ln ($\sigma_{\mu})$ represents the logarithm of the standard deviation of constant (subject-specific random effect; $\mu_{i}$);* p<0.1, ** p<0.05, *** p<0.01

NG: Nutri-Grade

**Table A3: Carryover Effect Test Results**

|  | Average NG | Sugar (g) per serving | Saturated fat (g) per serving | Calories (kcal) per serving |
| --- | --- | --- | --- | --- |
| NG | 0.19^***^ | -1.49^**^ | -0.0100 | -3.44 |
|  | [0.065, 0.31] | [-2.68, -0.31] | [-0.23, 0.21] | [-12.0, 5.16] |
|  |  |  |  |  |
| Sequence | 0.11 | -1.10 | 0.21 | 0.25 |
|  | [-0.11, 0.33] | [-3.14, 0.94] | [-0.13, 0.56] | [-12.7, 13.2] |
|  |  |  |  |  |
| Period | -0.097 | 0.36 | 0.14 | 3.70 |
|  | [-0.22, 0.028] | [-0.82, 1.54] | [-0.081, 0.35] | [-4.91, 12.3] |
|  |  |  |  |  |
| Constant | 2.61^***^ | 10.1^***^ | 0.66^***^ | 64.9^***^ |
|  | [2.43, 2.80] | [8.44, 11.8] | [0.37, 0.95] | [53.7, 76.1] |
| Observations | 269 | 269 | 269 | 269 |

Note: 95% confidence intervals are in brackets.* p<0.1, ** p<0.05, *** p<0.01

NG: Nutri-Grade
